# Microbial Reclamation of Chitin and Protein-Containing Marine By-Products for the Production of Prodigiosin and the Evaluation of Its Bioactivities

**DOI:** 10.3390/polym12061328

**Published:** 2020-06-10

**Authors:** Van Bon Nguyen, Dai Nam Nguyen, San-Lang Wang

**Affiliations:** 1 Division of Computational Mathematics and Engineering, Institute for Computational Science, Ton Duc Thang University, Ho Chi Minh City 700000, Vietnam; nguyenvanbon@tdtu.edu.vn; 2 Faculty of Applied Sciences, Ton Duc Thang University, Ho Chi Minh City 700000, Vietnam; 3 Department of Science and Technology, Tay Nguyen University, Buon Ma Thuot 630000, Vietnam; dainamnguyen.edu@gmail.com; 4 Department of Chemistry, Tamkang University, New Taipei City 25137, Taiwan; 5 Life Science Development Center, Tamkang University, New Taipei City 25137, Taiwan

**Keywords:** β-chitin, protein, prodigiosin, bioconversion, anticancer, AChE inhibitor, bioreactor

## Abstract

Chitin and protein-containing marine by-products (CPCMBPs), including crab shells, squid pens, and shrimp shells, were investigated as the sole carbon/nitrogen (C/N) source for prodigiosin (PG) production by *Serratia marcescens* TNU01 in a 250 mL Erlenmeyer flask and a 10 L bioreactor system. Among the used C/N source of CPCMBPs, squid pens powder (SPP) showed the most optimum PG productivity. Different ratios of chitin/protein combination were also used as the C/N sources for PG production. With a similar chitin/protein ratio (4/6) of squid pens, a significant PG productivity was achieved when the chitin/protein ratios were controlled in the range of 3/7–4/6. Maximum PG yield (3450 mg/L) by *S. marcescens* TNU01 was achieved in the bioreactor system containing 3 L medium of 1.75% SPP, 0.03% K_2_HPO_4_, and 0.05% MgSO_4_ at 25 °C for 12 h in dark. The results of in vitro bioassays reveal that the purified PG possesses acetylcholinesterase inhibitory activity and antioxidant as well as anticancer activities. This study suggests that squid pens may have the potential to be used for cost effective production of bioactive PG at a large-scale.

## 1. Introduction

Chitin and protein-containing marine by-products (CPCMBPs), such as squid pens, shrimp and crab shells, are obtained abundantly from the fishery processing industry [1,2,3,4,5,6]. CPCMBPs have high chitin content and were therefore utilized earlier for the production of chitin. CPCMBPs also contain a high amount of protein and mineral salts, thus, strong inorganic acid and alkali are used for demineralization and deproteinization of CPCMBPs to produce chitin, causing pollution due to acid or alkali liquid [4,7]. Bioprocessing, being environmentally-friendly, was established for conversion of CPCMBPs into chitin [8], and several other bioactive products were applied for nutritional, biotechnological, agricultural, biomedical, and pharmaceutical purposes [8,9,10,11,12,13,14,15]. Recently, CPCMBPs were explored for the production of antioxidant and anti-NO compound homogentisic acid [16,17] and extensively investigated for the biosynthesis of targets of enzyme inhibitors in antidiabetic drugs [1,16,18,19]. In this study, CPCMBPs were used for the cost effective production of bioactive prodigiosin (PG) at a high level via microbial fermentation.

PG is a bacterial secondary metabolite mainly produced by *Serratia marcescens* [11,20,21]. This red pigment has received much interest because of its vast array of potential effects such as antimicrobial, anticancer, antioxidant, antiprotozoal, insecticidal, and dyeing activities [11,21,22,23,24,25,26].

PG was recognized as a promising drug candidate, hence, there has been great interest in its mass production in significant amounts for further clinical evaluation [21]. To date, various types of commercially designed mediums have been reported for PG production by *S. marcescens* such as nutrient broth [27], tryptone/glycerol [28], Luria–Bertani (LB), tryptone yeast extract, tryptone soy, yeast malt, and glycerol extract broth [29], yeast extract [30], peptone glycerol broth [31], and 3-[*N*-morpholino]-ethanesulfonic acid [32]. For a lower cost of PG production, some low-cost starting materials have been searched for fermentation, including peanut seed, peanut oil, sesame seed, sesame oil, copra seed, coconut oil, corn steep, cassava, crude glycerol, the combination of corn steep/mannitol, cassava/mannitol, and peanut powder/olive oil/beef extract, and Luria–Bertani broth/sunflower oil [23,33,34,35,36,37]. For the multiple benefits of solving environmental problems and decreasing PG production costs, squid pens were used by *S. marcescens* TKU011 to produce PG with the productivity of 0.978 mg/mL [11], which increased up to 2.48 and 4.62 mg/mL after autoclave treatment [38] and optimization of chitin/protein ratio [39], respectively.

To achieve cost effective and large-scale production, this study used CPCMBPs as the sole carbon/nitrogen source for PG production by *S. marcescens* strains, which were isolated from the soils of the Central Highland of Vietnam. The optimized PG culture condition was achieved in scale-up production after investigating the effect of salts, chitin/protein ratio, light, and aeration. Finally, the PG produced by the bioreactor system was purified and utilized for the study of its potential biological activities.

## 2. Materials and Methods

### 2.1. Materials

*S. marcescens* TNU01, *S. marcescens* TNU02, and *S. marcescens* CC17 were newly isolated and identified based on the methods described earlier [39,40]. *S. marcescens* TKU011 was a stocked PG producing strain [11]. The CPCMBPs (crab shells, shrimp shells, and squid pens) were procured from Shin-Ma Frozen Food Co. (I-Lan, Taiwan). The demineralized crab shells powder (deCSP) and demineralized shrimp shells powder (deSSP) were produced as per the method reported by Wang et al. [41]. Shrimp heads powder (SHP) was procured from Fwu-Sow Industry (Taichung, Taiwan). The cancerous cell lines, including Hep G2, A549, WiDr, and MCF-7, were obtained from the Bioresources Collection and Research Centre (Hsinchu, Taiwan). *S. cerevisiae* α-glucosidase, rat α-glucosidase, *Bacillus subtilis* α-amylase, and porcine pancreatic α-amylase were purchased from Sigma Chemical Co. (St. Louis, MO, USA). All the other reagents and chemicals used were of the highest grade available.

### 2.2. Microbial Conversion for PG Production by S. marcescens

Production of PG by different *S. marcescens* strains using different C/N sources: Squid pens powder (SPP), the sole C/N source, was used for fermentation by various bacterial strains, including *S. marcescens* TNU01, *S. marcescens* TNU02, *S. marcescens* CC17, and *S. marcescens* TKU011. The cultivated medium with 40 mL (initial pH 6.15) in a 100 mL flask contained 1.5% of C/N source, 0.1% FeSO_4_(NH_4_)_2_SO_4_, and 0.1% K_2_HPO_4_. The cultivation was set at 25 °C for 2 d and shaking at 150 rpm. *S. marcescens* TNU01 was used as a potent PG-producing strain by fermenting SPP, SHP, deCSP, and deSSP under the same conditions as described above.

Investigation of the optimal ratio of chitin/protein combination: Chitin was isolated from squid pens following the method presented in the previous report by Wang et al. 2006 [42] in combination with casein in various ratios of 1/9, 2/8, 3/7, 4/6, 5/5, 6/4, 7/3, and 8/2 (chitin/protein) and fermented by *S. marcescens* TNU01 to investigate the optimal chitin/protein ratio. To compare the PG production effect of chitin and other polysaccharides (chitosan, starch, cellulose, and pectin), oligomers (dextrin and chitin oligomer) were combined with casein in the ratios of 3/7 and 4/6, then used for fermentation by *S. marcescens* TNU01.

Optimization of sulfate salts added to the culture medium: Several types of salts of sulfate, including K_2_SO_4_, MgSO_4_, FeSO_4_, (NH_4_)_2_SO_4_, ZnSO_4_, and CuSO_4_ were evaluated for optimization. The liquid medium (40 mL; initial pH 6.15) in a 100 mL flask containing 1.75% C/N source, 0.05% K_2_HPO_4_, 0.1% sulfate salt was used for cultivation at 25 °C for 2 d, at 150 rpm shaking. MgSO_4_ demonstrated the best effect and its added concentration was in the range of 0.01, 0.02, 0.03, 0.05, 0.1, 0.2, 0.3 and 0.4%, in combination with K_2_HPO_4_ at 0.05%, to be used as the basal salt added medium and the fermentation was performed as described above.

Optimization of phosphate salts added to the culture medium: Five types of phosphate salts, including KH_2_PO_4_, K_2_HPO_4_, NaH_2_PO_4_, Ca_3_(PO_4_)_2_, and Na_2_HPO_4_, were evaluated for optimization. In a 100 mL flask containing the 40 mL liquid medium (initial pH 6.15), 1.75% C/N source, 0.1% MgSO_4_, and 0.05% phosphate salt were cultivated at 25 °C for 2 d with at 150 rpm shaking. K_2_HPO_4_ demonstrated the best effect and its added concentration was in the range of 0.01, 0.02, 0.03, 0.05, 0.1, 0.2, and 0.4%, in combination with 0.1% MgSO_4_, to be used as the basal salt added medium and the fermentation was performed as described above.

The effect of several parameters of fermentation: Some parameters, such as fermentation temperature (20, 23, 25, 27, and 30 °C), the volume of air head space percentage (30, 40, 50, 60, 70, and 80%) in a 100 mL flask, shaking speed (0, 50, 100, 150, and 200 rpm), initial pH of liquid medium (pH 5.15–9.65), cultivation time (0–4 d), and light (in dark and in light), were tested for their effect on PG production. The most optimal cultivation conditions in the flask were used for the fermentation in the bioreactor system BioFlo/CelliGen 115 (Eppendorf North America, Connecticut, US) (3 L liquid medium in the 10 L bioreactor system) at different periods of time (4, 8, 12, and 16 h). Simultaneously, fermentation was also conducted in the flask at the same conditions in the fermentation time from 4 to 48 h for comparison.

### 2.3. The Quantitation and Purification of PG Produced by S. marcescens TNU01

The method mentioned in a previous study [11] was used here for determination of PG content. Methanol was mixed with fermented medium broth in the volume of 4 and 0.5 mL, respectively, and 0.5 mL of 2% (*w*/*v*) AlK(SO_4_)_2_·12H_2_O was added, mixed, and centrifuged for 5 min at 1400× *g* for harvesting the supernatant. This supernatant and 0.5 N HCl in methanol were mixed in the ratio of 0.5/4.5 and used for the detection of optical density at 535 nm (OD_535nm_). The purified PG obtained from the previous study [39] was used as the standard for conversion OD_535nm_ into PG content.

PG extraction was carried out according to the previously described method [11]. The culture broth was centrifuged for 15 min at 10,000× *g* to obtain the supernatant, which was then mixed with ethyl acetate (EA) in an equal volume and kept in a funnel with shaking every 30 min for 3 h. The ethyl acetate layer containing PG was collected. Acetone was mixed with the cell pellets containing PG to dissolve PG and this solution was then centrifuged for 15 min at 10,000× *g* for removing the cell pellets residue to harvest acetone solution containing PG. The solutions of acetone and EA containing PG were mixed together then dried to crude PG powder by evaporation of the solvent and oven air drying at 55 °C. The silica column (Geduran^®^ Si 60, size: 0.040–0.063 mm; Merck KGaA, Darmstadt, Germany) was used for the first step of PG purification. This crude PG was further purified by TLC (thin layer chromatography) separation. The PG lane on the TLC plate was cut into thin pieces and methanol was added to dissolve PG. Then, pure PG was obtained by evaporating the solvent in a rotary evaporator (IKA, Staufen, Germany) at 55 °C under vacuum. The purified PG was used for determination of UV (FP-8200 Fluorescence Spectrometer, Jasco International Co., Ltd., Tokyo, Japan) and mass spectrometry (Bruker Daltonics, Bremen, Germany), and evaluation of biological activities.

### 2.4. Bioactivities Assays

The cancerous cell lines A549, Hep G2, WiDr, and MCF-7 were used to evaluate the anticancer effect of PG following the method described in detail in the previous study [43]. Acetylcholinesterase inhibition was measured using the method reported by Tan et al., 2018 [44], and alpha-amylase and α-glucosidase inhibition were determined following the detailed methods mentioned by Nguyen et al., 2017 [45], and Nguyen et al., 2018 [46], respectively. DPPH radical scavenging capacity assay was determined according to the method mentioned in the previous report by Nguyen et al., 2018 [16].

## 3. Results and Discussion

### 3.1. Production of PG by Different S. marcescens Strains Using Various C/N Sources

**Figure 1:**
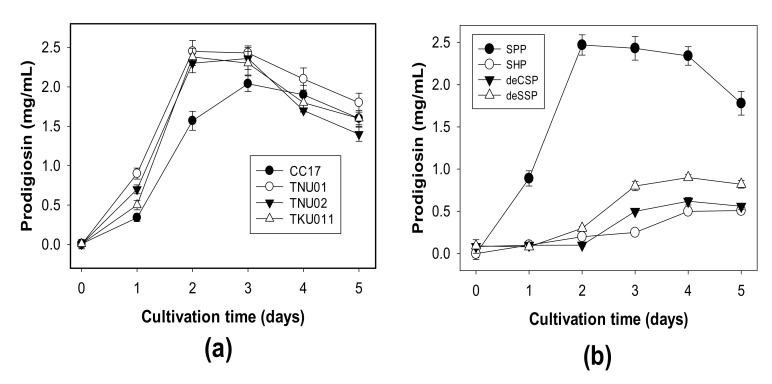
PG production by different *S. marcescens* strains, including *S. marcescens* TNU01, *S. marcescens* TNU02, *S. marcescens* CC17 and *S. marcescens* TKU011 (**a**), and the use of different chitin and protein–containing marine by–products for PG production by *S. marcescens* TNU01 (**b**). SPP: squid pens powder, SHP: shrimp head powder, deCSP: demineralized crab shells powder, deSSP: demineralized shrimp shells powder.

Squid pens powders were used for fermentation by different *S. marcescens* strains to compare their PG producing ability. As shown in Figure 1a, all four tested strains showed high potential in PG production at a scale of 2.04–2.43 mg/mL on day 3 of fermentation. Strains TNU01, TNU02, and TKU011 could reach the highest PG yield production on day 2. Of these, strain TNU01 showed the highest PG yield of 2.45 mg/mL and was used for further investigations. *S. marcescens* TNU01 was evaluated for its ability to ferment various kinds of CPCMBPs. As presented in Figure 1b, *S. marcescens* TNU01 could convert all kinds of CPCMBPs into PG. Among them, the most suitable C/N source was SPP, with the highest yield of 2.47 mg/mL PG in the shortest fermentation time (2 days).

**Figure 2:**
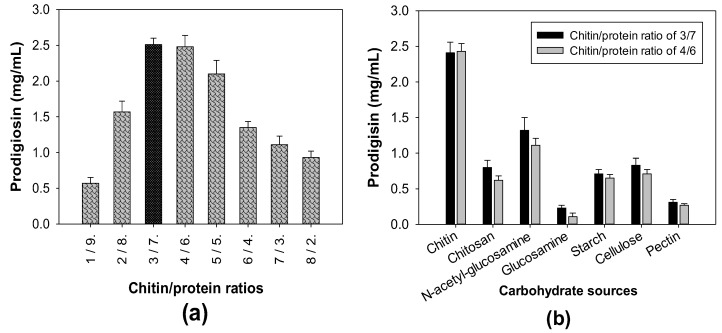
The influence of chitin/protein ratio (**a**) and various carbon sources (**b**) on PG biosynthesis by *S. marcescens* TNU01. A carbohydrate/protein ratio of 3/7 or 4/6 was used for fermentation.

Squid pens, one of the CPCMBPs, have been reported to possess significant contents of chitin and protein at approximate values of 40% and 60% of the total content, respectively [17]. A suitable ratio of chitin and protein combination exhibited a significant effect on PG production by *S. marcescens* [39]. To explore the optimal combination ratio, the chitin extracted from squid pens was combined with free protein in various ratios and used for cultivation by *S. marcescens* TNU01 to produce PG. The results presented in Figure 2a indicate that the combination of chitin and protein in the ratios of 3/7 and 4/6 were optimal for *S. marcescens* TNU01-induced highest PG productivity of 2.51 and 2.48 mg/mL, respectively. Interestingly, the chitin and protein existed in SPP in an approximate ratio of 4/6 (40%/60%), thus, SPP was solely used as the substrate to be fermented by *S. marcescens* TNU01 for cost effective PG biosynthesis. To compare the effect on enhancing PG production of SPP chitin, its derivative (chitosan), monomers (glucosamine, *N*-acetyl-glucosamine), and some other polysaccharides (starch, cellulose, and pectin), these carbon sources were combined with free protein and used for fermentation by *S. marcescens* TNU01. Among tested carbon sources, SPP chitin demonstrated the best effect on PG production by *S. marcescens* TNU01 with the highest yield (2.41–2.43 mg/mL), following by its monomer, *N*-acetyl-glucosamine (1.11–1.32 mg/mL), and then, other carbon sources (≤0.83 mg/mL) (Figure 2b).

### 3.2. The Effect of Addition of Sulfate and Phosphate Salts to the Culture Medium and Enhancement of PG Production by Optimizing Some Parameters

**Figure 3:**
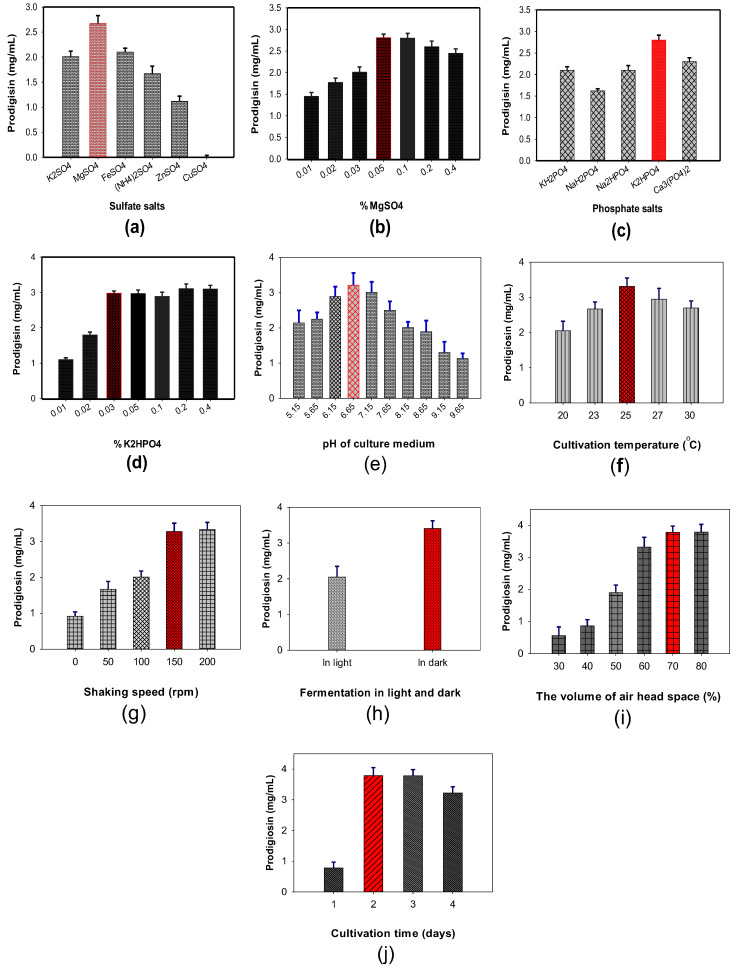
The effect of sulfate salts (**a**), added MgSO_4_ (**b**), phosphate salts (**c**), and added K_2_HPO_4_ (**d**), initial pH of the medium (**e**), cultivation temperature (**f**), shaking speed (**g**), fermentation in light (no cover the flask) or in dark (cover the flask) (**h**), the volume of air headspace percentage (**i**), and cultivation time (**j**) on PG production by *S. marcescens* TNU01.

Sulfate and phosphate salts added to the culture medium demonstrated a significant effect on PG production by *S. marcescens,* however, different strains may require different sources for the optimal supply of sulfate and phosphate [11,39,47]. Thus, the evaluation of a suitable salt source for maximal PG production is in need. Different sulfate salts such as K_2_SO_4_, MgSO_4_, FeSO_4_, (NH_4_)_2_SO_4_, ZnSO_4_, and CuSO_4_ and phosphate salts, including KH_2_PO_4_, NaH_2_PO_4_, Na_2_HPO_4_, K_2_HPO_4_, and Ca_3_PO_4_ were added to the culture medium to investigate their effect on PG production by *S. marcescens* TNU01. As mentioned in Figure 3, MgSO_4_ demonstrated a significant enhancement of PG production (2.67 mg/mL) compared to other salts (0–2.1 mg/mL) (Figure 3a) and 0.05% MgSO_4_ added to the culture medium was observed to be the optimal concentration for achieving highest PG productivity (Figure 3b). Among the phosphate salts, the most suitable candidate was K_2_HPO_4_ (Figure 3c) at 0.03%, resulting in the maximum PG yield production of 2.98 mg/mL (Figure 3d).

To harvest max PG yield by *S. marcescens* TNU01 fermentation, we also investigated the optimal concentrations of some parameters, including initial pH of the medium (Figure 3e), cultivation temperature (Figure 3f), shaking speed (Figure 3g), light (in dark and in light, Figure 3h), the volume of air headspace percentage in a 100 mL flask (Figure 3i), and cultivation time (Figure 3j). Overall, the production of PG by *S. marcescens* TNU01 achieved the maximal yield (3.79 mg/mL) in a 30 mL culture medium with initial pH of 6.65, containing 1.75% SPP, 0.03% K_2_HPO_4_, 0.05% MgSO_4_. The cultivation process was carried out at 25 °C, 150 rpm (shaking speed), 70% of air headspace, at 150 rpm shaking, in dark in 2 d. Notably, the percentage of air headspace was found as a significant factor in PG biosynthesis by *S. marcescens* was observed for the first time in this study.

### 3.3. Scale-Up of PG Production in A Bioreactor System

**Figure 4:**
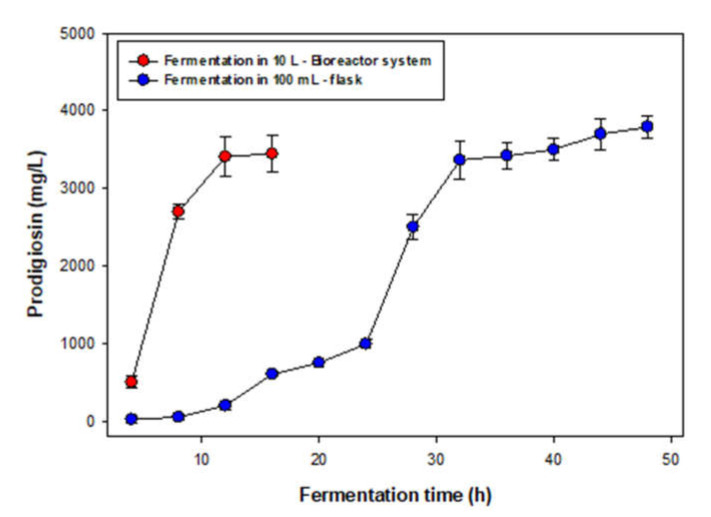
PG production by *S. marcescens* TNU01 in 10 L bioreactor systems and in a 100 mL-flask. An amount of 300 mL of seed bacteria was prepared in a flask for 1.5 days and injected in 10L bioreactor systems containing 3 L of liquid medium with other optimized compositions of other parameters as obtained from Section 3.2. The medium was sampled, and PG was detected from 4 to 16 h of fermentation. PG production also by *S. marcescens* TNU01 in optimal conditions in a 100 mL flask.

After achieving an optimal condition for PG production at a minor-scale, for achieving large-scale PG production, a 10 L bioreactor system was used. The PG yield was detected in the period of fermentation from 4 to 16 h (Figure 4). For comparison, fermentation in a 100 mL flask was also carried out (4–48 h). The results presented in Figure 4 indicated that PG production by *S. marcescens* TNU01 in bioreactor systems showed good results with comparable PG yield (3450 mg/L) to that of PG production in a flask (3790 mg/L), and achieved large-scale PG production in 3L fermentation per time in one pilot bioreactor, in a short time of fermentation (12 h). To date, various PG production systems have been reported, although, only a few studies have reported large-scale PG production in a 10 L bioreactor system for saving fermentation time and large-scale PG biosynthesis.

### 3.4. Isolation and Qualification of PG from Fermented Culture Broth

**Figure 5:**
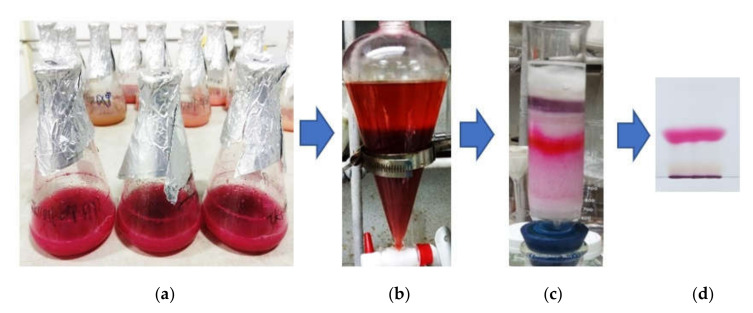
The process of PG purification. The liquid culture medium fermented by *S. marcescens* TNU01 under optimal condition (**a**) was centrifuged to obtain the supernatant containing PG, which was primarily extracted by ethyl acetate (**b**). The crude PG containing in the ethyl acetate layer was next separated on a silica gel column (**c**) and finally, isolated as a pure compound by TLC separation (**d**).

The extraction of PG was performed following the process reported earlier by Wang et al., 2012 [11]. The two portions, including that of the ethyl acetate layer and the acetone extract containing PG of supernatant and cell pellet, respectively, were mixed and dried to powder. This crude PG powder was primarily separated via a silica open column, then isolated as a pure compound by using TLC separation. The purification process of PG is illustrated in Figure 5.

**Figure 6:**
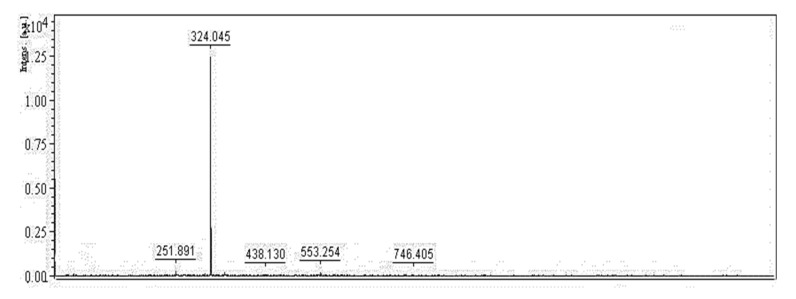
Mass of purified PG was detected by MALDI-TOF MS spectrum. A matrix, including 2,5-dihydroxybenzoic acid in TFA-H_2_O-CAN (0.1/50/50%, *v*/*v*/*v*, respectively) solution was used to prepare the sample. The prepared sample was analyzed by MALDI-TOF using a nitrogen laser generator emitting at 337 nm in a linear mode. For each spectrum, the data of 30–50 laser shots were acquired and analyzed.

**Figure 7:**
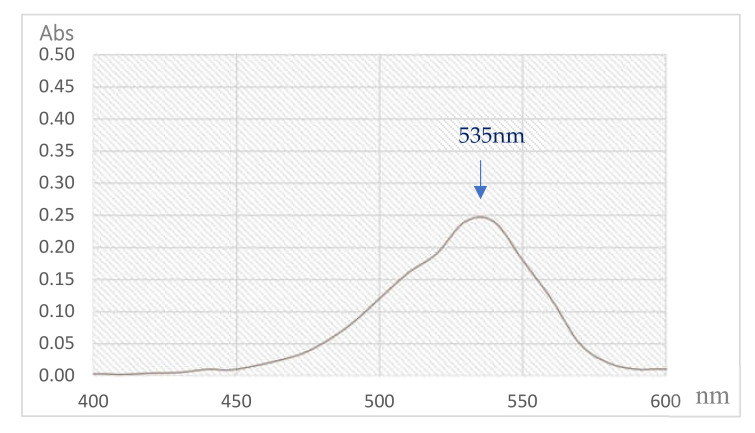
The spectrum of UV absorption of purified PG produced by *S. marcescens* TNU01 under optimal condition.

PG, a secondary metabolite is mainly produced by the bacterium *S. marcescens* [21]. The red pigment synthesized by *S. marcescens* TNU01 extracted and purified in this study was reconfirmed as PG through some rapid techniques, including its mass and UV absorption. As shown in Figure 6 and Figure 7, this purified compound exhibited mass (M + 1) of 324.045, and maximum UV absorption at 535 nm, concurrent with the specific mass and UV absorption of PG [11,39]; therefore, this purified pigment was determined as PG.

### 3.5. Evaluation of Biological Activities of PG

**Figure 8:**
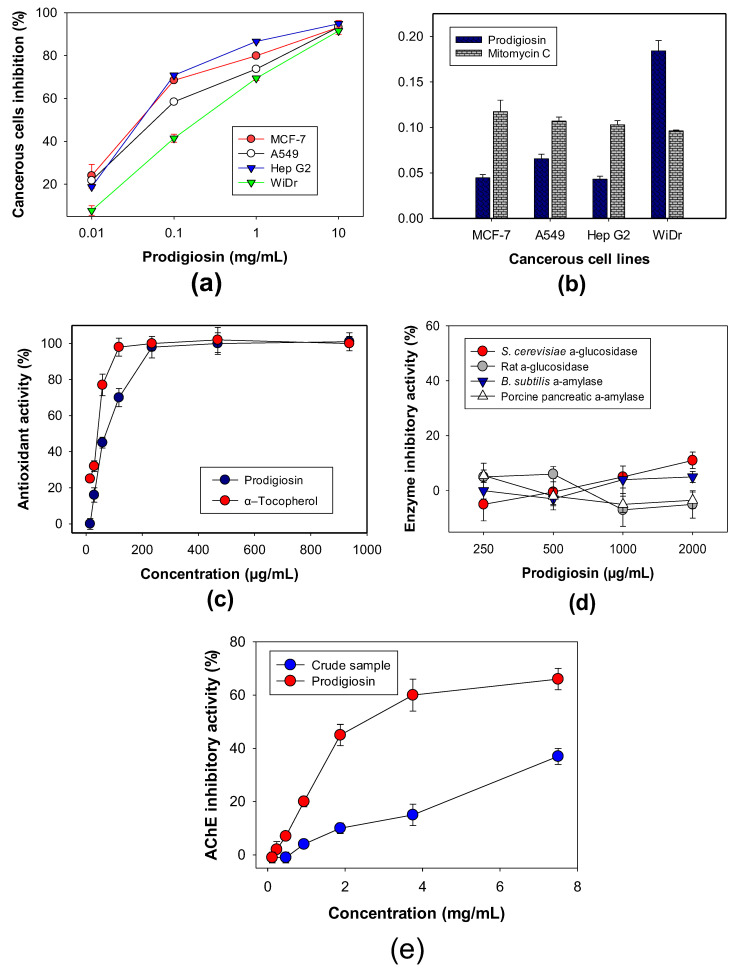
Biological activities of PG, including anticancer activity (**a**,**b**), antioxidant activity (**c**), enzyme inhibitory activity target in anti-diabetes (**d**), and AChE inhibitory activity: Acetylcholinesterase inhibitory activity (**e**).

PG has been reported to exhibit vast arrays of potential bioactivities [11,21,22,23,24,25,26,48,49,50,51,52]. To confirm and investigate the biological efficacy of the newly purified PG in this study, several bioactivities, including anticancer effect, antioxidant activity, and enzymes inhibition targets in anti-diabetes, and anti-Alzheimer were assessed and the results were recorded and illustrated in Figure 8.

Some cancerous cell lines, such as Hep G2, A549, WiDr, and MCF-7, were used to evaluate the anticancer activity of PG (Figure 8a), which showed a strong inhibitory effect on the four tested cell lines with high maximum inhibitory activity (≥90%) at 10 µg/mL. For clarification of the anticancer effect of PG, the activity was also calculated and expressed in terms of IC50 values, which is defined as the concentration of a compound that reduces 50% of cancerous cells compared to the control group under the tested condition; the lower IC50 values the compound possesses, the stronger is the activity. Among the four tested cancerous cell lines, PG demonstrated potential inhibitory activity against Hep G2, A549, and MCF-7, and a moderate activity against WiDr with low IC50 values of 0.0432, 0.0655, 0.0446, and 0.1842 µg/mL, respectively (Figure 8b). In comparison, mitomycin C, a positive anticancer agent, was also tested for its anticancer activity with the IC50 values of 0.10296, 0.1070, 0.1174, and 0.0961 µg/mL, respectively, against Hep G2, A549, and MCF-7, and WiDr. Therefore, PG demonstrated stronger inhibitory activity against Hep G2, A549, and MCF-7 than that by mitomycin C. PG has been widely investigated for its potent anticancer effect on various cancerous cells. However, only few data of anti-WiDr of PG have been reported [39].

Antioxidants have been believed to terminate the harmful property of free radicals that causes damage to proteins, lipids, and the DNA of cells, leading to various diseases [53]. In the current report, we used DPPH radical scavenging capacity to evaluate the antioxidant effect of PG. α-Tocopherol was used as a positive antioxidant. As shown in Figure 8c, PG exhibited a moderate antioxidant activity, which reached up to 98% at its tested concentration of 234 µg/mL, comparable to α-tocopherol (98%) at 117 µg/mL (Figure 8c). The antioxidant capacity of PG and α-tocopherol was also expressed as IC50 values of 79.1 and 38.5 µg/mL, respectively. PG has been reported to show great DPPH radical scavenging capacity of 86% and 99% [54,55], but with no reports on its IC50 value. However, there are rare reports of DPPH radical scavenging capacity of PG beyond the above-cited papers; as such, the result in this study contributes to reconfirm and show in detail the DPPH radical scavenging activity of PG, and also indicates that PG may be a good candidate as an anti-radical scavenging compound.

PG was reported showing antidiabetic effect by anti-insulitis [56]. To investigate whether the antidiabetic activity of PG is linked to enzyme inhibition mechanism, various enzyme targets in type 2 diabetes, including *S. cerevisiae* α-glucosidase, rat α-glucosidase, *B. subtilis* α-amylase, and porcine pancreatic α-amylase, were evaluated. However, no inhibitory effect of PG against any tested enzymes was observed (Figure 8d). In summary, PG showed its inhibitory activity against acetylcholinesterase, as an anti-Alzheimer molecule with a moderate max inhibitory value of 62%, and an IC50 value of 1.12 mg/mL. The crude sample was also tested and showed a lower inhibition value of 37% (Figure 8e). Notably, this in vitro anti-Alzheimer property of PG was a new observation to be reported for the first time in this study.

## 4. Conclusions

The chitin and protein-containing marine by-product squid pens were cost effectively bioprocessed into PG via *S. marcescens* TNU01 fermentation. The PG production was achieved in a large-scale (3 L) 10 L bioreactor system with the PG yield of 3450 mg/L by application of the newly designed liquid medium with 1.75% SPP, 0.03% K_2_HPO_4_, and 0.05% MgSO_4_ at 25 °C for 12 h in dark. The PG was purified and investigated for its potential anticancer activity, moderate antioxidant effect, and novel moderate acetylcholinesterase inhibitory activity. The results obtained in this study suggest that squid pens may be used for cost effective large-scale production of bioactive PG.
